# Harnessing Unconventional T Cells for Immunotherapy of Tuberculosis

**DOI:** 10.3389/fimmu.2020.02107

**Published:** 2020-09-03

**Authors:** Marco P. La Manna, Valentina Orlando, Bartolo Tamburini, Giusto D. Badami, Francesco Dieli, Nadia Caccamo

**Affiliations:** ^1^Central Laboratory of Advanced Diagnosis and Biomedical Research, Palermo, Italy; ^2^Department of Biomedicine, Neurosciences and Advanced Diagnostics, University of Palermo, Palermo, Italy

**Keywords:** host-directed therapy, tuberculosis, unconventional T cells, cytotoxicity, T cell receptor

## Abstract

Even if the incidence of tuberculosis (TB) has been decreasing over the last years, the number of patients with TB is increasing worldwide. The emergence of multidrug-resistant and extensively drug-resistant TB is making control of TB more difficult. *Mycobacterium bovis* bacillus Calmette–Guérin vaccine fails to prevent pulmonary TB in adults, and there is an urgent need for a vaccine that is also effective in patients with human immunodeficiency virus (HIV) coinfection. Therefore, TB control may benefit on novel therapeutic options beyond antimicrobial treatment. Host-directed immunotherapies could offer therapeutic strategies for patients with drug-resistant TB or with HIV and TB coinfection. In the last years, the use of donor lymphocytes after hematopoietic stem cell transplantation has emerged as a new strategy in the cure of hematologic malignancies in order to induce graft-versus leukemia and graft-versus-infection effects. Moreover, adoptive therapy has proven to be effective in controlling cytomegalovirus and Epstein-Barr virus reactivation in immunocompromised patients with *ex vivo* expanded viral antigen-specific T cells. Unconventional T cells are a heterogeneous group of T lymphocytes with limited diversity. One of their characteristics is that antigen recognition is not restricted by the classical major histocompatibility complex (MHC). They include CD1 (cluster of differentiation 1)–restricted T cells, MHC-related protein-1–restricted mucosal-associated invariant T (MAIT) cells, MHC class Ib–reactive T cells, and γδ T cells. Because these T cells are genotype-independent, they are also termed “donor unrestricted” T cells. The combined features of low donor diversity and the lack of genetic restriction make these cells suitable candidates for T cell–based immunotherapy of TB.

## Introduction

Tuberculosis (TB) is the deadliest infectious disease worldwide, even if the global incidence has declined over the past decades. The etiologic agent *Mycobacterium tuberculosis* still causes more than 10 million cases and 1.5 million deaths every year. Although drug treatment usually provides microbiological cure in patients treated with 6-month regimen for drug-sensitive strains, 1.1 million people remain sick (1), because of the spread of strains resistant to multiple drugs. Moreover, it is estimated that one-quarter of people worldwide are latently infected, and of these, 5 to 15% will develop TB during their lifetimes, due to the higher risk for people with immunocompromised system, such as human immunodeficiency virus (HIV), malnutrition, or diabetes, or people who use alcohol or tobacco (2). Treatment for latently infected people is necessary for the global control of TB. The emergence of multidrug-resistant TB remains a growing threat to global public health; in fact, in the absence of a vaccine more efficient than *Mycobacterium bovis* bacillus Calmette–Guérin (BCG) vaccine to prevent primary infection or progression to active TB in latently infected people, TB global control needs novel therapeutic strategies in order to improve *M. tuberculosis* eradication and limit the excessive pathology.

In this context, the research of more effective and cheaper drugs represent one of the solutions (3, 4), while therapeutic interventions that can modulate the immune response have been proposed (5–7).

These interventions, termed “host-directed therapies” (HDTs), are directed to evaluate different aspects in order to better understand the inflammatory and immune pathways governing protective or detrimental outcomes of the disease. HDTs consider several mechanisms of action: the research of biological drugs useful to reduce treatment regimens strategy to reduce TB pathology targeting *M. tuberculosis* such as granuloma structure, autophagy induction, anti-inflammatory response, and cell- and antibody-mediated immune responses (8–10).

We review here developments and current advances in adoptive T cell therapy; in particular, we will focus on the role of unconventional T cells and discuss whether such approach may be helpful to offer a valid strategy for the cure of TB applicable also to other infectious diseases.

As the role of CD4 and CD8 T cells has been largely studied in TB, highlighting the limit of the high most polymorphic presentation of peptides antigens by MHC classes I and II molecules, the donor unrestricted nature of antigen presentation by molecules that are apparently non-polymorphic, elicits strong interest for vaccine or T cell immunotherapeutic approaches to target the entire global population without respect to host genetic factors.

## Natural Killer T and Mucosal-Associated Invariant T Cells

Natural killer T (NKT) and MAIT cells constitute a subset of T cells that recognize antigens of non-peptidic nature. These cells are named as unconventional or “innate-like” T cells for their distinct features (11, 12). These cells have different memory, kinetics, and ligand recognition compared to conventional T cells (13).

**FIGURE 1:**
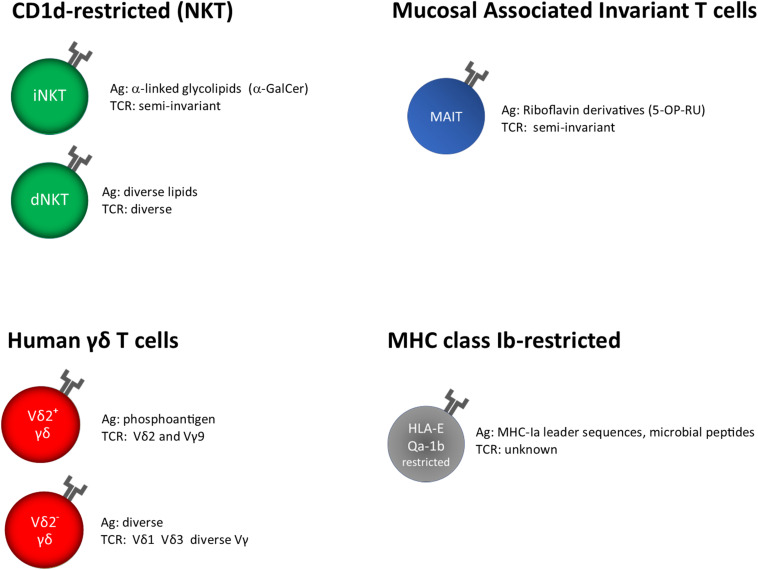
Unconventional T cells, grouped on the base of their restriction elements. α-GalCer, α-galactosyl ceramide; 5-OP-RU, 5-(2-oxopropylideneamino)-6-D- ribitylaminouracil; unknown, insufficient or very limited data.

MAIT and NKT cells recognize microbial metabolites and lipids presented by MHC-related protein 1 (MR1) and cluster of differentiation 1d (CD1d), respectively (Figure 1).

In *M. tuberculosis* infection, the role of NKT cell subsets has been investigated; here, we report some evidences of their role depending on the type of mycobacterial antigens specifically recognized.

### NKT Cells

It has been shown that NKT cells play a key role in a variety of infectious and autoimmune diseases and cancer (14). NKT cells express a rearranged αβ T cell receptor (TCR) and NK cell receptors, which confer the capability to exert several effector functions in immune surveillance. Based on their TCR repertoire and antigen recognition, NKTs can be split up into invariant (iNKT) and diverse (dNKT). Both cell types are CD1d-restricted and respond to glycolipid and lipid antigens/CD1 complexes, respectively.

### Invariant NKT

Invariant NKT cells, also termed classical type I cells, use an invariant TCRα chain (Vα14-Jα18 in mice, Vα24-Jα15 in humans) paired with limited TCRβ chains (Vβ7, 8.2 or 2 in mice, Vβ11 in humans), and constitute a majority of the overall CD1d-restricted repertoire (15, 16). iNKT cells specifically recognize the endogenous α-linked monoglycosylceramides, α-galactosyl ceramide (αGalCer) and α-glucosylceramide (αGalcCer). Similar to helper CD4 T cells, notwithstanding their specificity for αGalCer recognition, they are able to produce different cytokines such as interferon γ (IFN-γ), interleukin 4 (IL-4), or IL-17A, which designate them as iNKT1, iNKT2, and iNKT17.

### Diverse NKT Cells

Diverse NKT cells have a more diverse repertoire and recognize a wider range of self and non-self lipids such as sphingolipids (e.g., sulfatides and βGalCer) and phospholipids (e.g., phosphatidylinositol, phosphatidylglycerol, and phosphatidylethanolamine).

Mycolipids, lipids belonging to the mycobacterial cell wall, can bind CD1d molecules and activate human NKT cells. dNKT cell activation in responses to different antigens has been detected in individuals infected with *M. tuberculosis* (17).

Their role has been investigated in *in vitro*, *in vivo*, and in preclinical models of *M. tuberculosis* infection (18–22). Active TB patients had a decreased percentage of iNKT cells in peripheral blood or bronchoalveolar lavage samples (23, 24), with respect to subjects with latent TB infection (25–30), even if these cells still maintained the capability to secrete high amounts of IFN-γ and displayed an activated phenotype (30, 31).

However, NKT cells from active TB patients express programmed death 1 (PD-1) molecule at cell surface that leads to their subsequent apoptosis, an event that can be abrogated through PD-1 blockade (23, 32). This finding is highly suggestive of an immunological approach to achieve a protective immune response against *M. tuberculosis*. In addition, iNKT cells present in pleural effusion in TB patients produce IL-21 and can then participate to local B cell activation and humoral immune response to *M. tuberculosis* (33).

While these studies do not clarify a key role for NKT cells in human TB, their rapid activation and the different role they can exert in TB infection poise them as an intriguing target for cell-based therapies (34).

Finally, results from non-human primate (NHP) models of *M. tuberculosis* infection have demonstrated that CD8^+^ iNKT cells play a protective role in preventing lung pathology (19, 35).

Altogether, these cells are promising tools for potential use as HDT in human TB, in a similar way to their use in cancer immunotherapy (36).

### MAIT Cells

MAIT cells recognize very rapidly non-peptidic antigens presented by MR1 molecule (37). MAIT cells respond to Ag stimulation rapidly either because they are constitutively activated by Ags derived from commensal bacteria and maintain activated/memory phenotype, or their clonal size is larger than conventional T cells (38). In humans, MAIT cells express a Vα7.2-Jα33/12/20, TCRα chain preferentially paired with Vβ2 or Vβ13 (39–41). In their structure and function, MAIT cells represent a bridge between the innate and adaptive immunity. They are αβ T cells whose TCRs have restricted diversity and recognize small microbial metabolites. In the riboflavin synthesis process, many bacterial and fungal organisms produce small intermediates able to activate MAIT cells (42–45). There is also evidence that non–riboflavin-based antigens, also of microbial origin (46) and tumor cell–derived molecules (47), can bind to MR1 and activate some MR1-restricted T cells, although the identity of these antigens remains to be defined. MAIT cells comprise 1 to 10% of circulating CD3^+^ T cells in healthy adults (48, 49) and have also been found in the gastrointestinal tract, liver, and airways (50–54).

The role of this population of T cells in the response against microorganisms is still a matter of studies. TCR-mediated activation of MAIT cells leads to cytokines production, cytotoxic effector function, migration, and proliferative expansion (12, 39, 55).

Because MAIT cells can respond to a range of bacteria and yeasts, several studies have supported the proposal of a peculiar and non-redundant role in protection against infectious diseases.

In particular, MAIT cells can contribute to the destruction of infected cells and activation of other immune cell types (56) through the release of perforin and granzymes; moreover, they are a source of several proinflammatory cytokines and chemokines, such as tumor necrosis factor α (TNF-α), IFN-γ, IL-17A, and MIP-1α. MAIT cells can also produce IL-22, IL-13, IL-26, or IL-2, depending on the different cytokine milieus and tissue localization (57–60). Indeed, MAIT cells can even be activated by inflammatory stimuli in the absence of TCR-mediated antigen recognition (57–59, 61–68).

In animal models of mycobacterial infection, MAIT cells are able to reduce mycobacterial burden and increase the ability of macrophages to inhibit the growth of intracellular bacilli (56). MAIT cells were demonstrated to protect mice against mycobacterial infection (55, 69).

In human TB infection, several studies have evaluated MAIT cell frequencies in peripheral blood or in inflamed tissues, demonstrating that this cellular population decreases during active TB in diverse geographic settings (55, 70–72). Moreover, this decrease is paralleled by their enrichment in the inflamed tissue such as that found in the lungs and pleural effusions of TB patients (73), where they displayed a phenotype of activated/memory cells and a higher capacity to produce cytokines such as IFN-γ and TNF-α (74).

Studies have evaluated the relative increase or decrease of the absolute number or percentage with the progression of TB infection. In fact, in some circumstances, MAIT cell deficiency has been associated with the different clinical TB conditions. Moreover, their decrease has been inversely correlated with other biological parameters, such as high levels acid–fast bacilli in sputum of TB patients or with systemic markers of inflammation (72). Additionally, peripheral blood MAIT cells showed an impaired production of cytotoxic molecules and cytokines such as IFN-γ in patients with active pulmonary TB (75). These functionally impaired MAIT cells express higher levels of proapoptotic markers and PD-1 than MAIT cells from non-TB patients (71, 72, 74). PD-1^+^ MAIT cell expression has been related to active TB status and declines with TB treatment (74, 76).

*In vitro* blockade of PD-1 can increase IFN-γ production in circulating MAIT cells from patients with TB, thus resembling the data obtained in studies on NKT cells.

Human data about MAIT cells still gave incomplete information about the role these cells play in bacterial disease, but it was stated that in active TB disease MAIT cells decrease in peripheral blood and those that remain show a dysfunctional, yet reversible phenotype. An immunological approach could be to use MR1 ligands in therapeutic settings, in order to potentiate the functional activities of MAIT cells in *M. tuberculosis* infection. This therapeutic approach would be applicable in the heterogeneous human populations because of the monomorphic nature of MR1, which displays very limited restriction barrier in human populations.

### γδ T Cells

T cell receptor (TCR) of γδ T cells in humans consists of a γ and a δ chain; they are usually identified on the base of the δ chain expressed on the surface, Vδ1^+^, Vδ2^+^, and a minor subset Vδ3^+^. The knowledge about their antigen recognition repertoire is not fully elucidated; in fact, some TCRs specifically recognize soluble antigens in the absence of Ag presentation, whereas other can bind antigens presented by MHC-I like molecules such as MICA, CD1, and EPCR (77, 78) (Figure 1).

The Vδ2^+^ T cells are the major γδ subset in the blood, and their TCR consists of a Vδ2 and a Vγ9 chain that recognize microbially derived phosphorylated antigens associated with the monomorphic butyrophilin 3A1 (BTN3A1) molecule (79). Metabolites known as phosphoantigens (PAgs) activate Vγ9Vδ2 T cells (80, 81). One of these metabolites is isopentenyl pyrophosphate (IPP), produced in eukaryotes through the mevalonate pathway, a pathway involved in protein prenylation, and in cholesterol synthesis (82). A dysfunction of this pathway can lead to overproduction of endogenous IPP, as occurs in stressed cells (83, 84). Moreover, some drugs can manipulate the production of endogenous IPP. Another metabolite able to activate Vγ9Vδ2 T cells is hydroxymethyl-but-2-enylpyrophosphate (HMBPP), an intermediate of the alternative, non-mevalonate pathway of cholesterol used by some Eubacteria and by *Plasmodium falciparum*, the etiologic agent of malaria (85, 86).

Pags are recognized in a TCR-dependent manner, and very recently, the molecule BTN3A1, belonging to the butyrophilin (BTN) protein family, has been implicated as essential molecule in the PAgs activation pathway of Vγ9Vδ2 T cells (87–89).

BTN3A proteins are receptors expressed in several cell types, including immune cells and some malignant cells such as ovarian cancer. Even if the prominent role of BTN3A1 in PAg-induced Vγ9Vδ2 T cell activation is well documented, the mechanism of recognition of the PAg has not been fully delineated. There is strong evidence that Vγ9Vδ2 T cell activation is due to an intracellular sensing of PAg through the interaction with the B30.2 domain of BTN3A1 molecule (90–93).

Vγ9Vδ2 T cells can recognize another antigen belonging to *M. tuberculosis*, the 6-*O*-methylglucose-containing lipopolysaccharides.

Even if other two minor subsets of γδ T cells are less represented in blood, they are able to recognize mycobacterial lipid or glycolipids antigens presented by CD1c and CD1d molecules. Interestingly, some Vδ1^+^γδ T cells are activated following recognition of α-GalCer presented by CD1d molecule (94), which might be relevant in human clinical trials.

Generally, any γδ T cell antigen could be used to design TB vaccines due to their ability to drive *in vivo* expansion of *M. tuberculosis*–reactive γδ or to generate *in vitro* γδ T cells to be used in adoptive cell therapy.

In human blood, γδ T cells are relatively abundant, so it is possible to isolate them in large numbers and characterize their effector mechanisms such as the ability to produce T_H_ 1-, T_H_ 2-, or T_H_17-type cytokines and to exert potent cytotoxicity, this latter function being closely correlated to the elimination of infected or tumoral cells. These findings, together with their capacity to rapidly migrate to peripheral sites suggest that γδ T cells are good candidates for adoptive transfer models of therapy.

They are included in the cluster of unconventional T cells, because of their MHC unrestricted antigen recognition; therefore, they have been used and transferred from an MHC mismatched background without causing graft-versus-host disease (GVHD), which can be a life-threatening complication with adoptive transfer of conventional αβ T cells (95).

In *M. tuberculosis* infection, Vγ9Vδ2 T cells are activated rapidly after PAg stimulation, exerting effector mechanisms such as release TNF-α and IFN-γ (96), and cytotoxic molecules such as perforin, granzymes, and granulysin that are involved in the killing of *M. tuberculosis*–infected macrophages and in the reduction of the viability of intracellular and extracellular *M. tuberculosis* (97). In humans, BCG vaccination determines *in vivo* expansion of γδ T cell population with a memory phenotype, and similar findings have been observed in BCG or *M. tuberculosis*–infected rhesus macaques.

Moreover, adoptive transfer of activated Vγ9Vδ2 T cells in *M. tuberculosis*–infected macaques has demonstrated protection against this intracellular pathogen (79, 96). The adoptive transfer of γδ T cells in NHP reduced the bacterial burden and limited disease to the infected lobe by prevention of dissemination (98). In primates, this expansion is clonal and selects for Vγ9^+^Vδ2^+^ TCR usage (79). This clonal expansion is due to the specific recognition of PAgs conserved among mycobacteria (99), recognized by butyrophilin-dependent manner (96, 100). Further advances in the use of γδ T cells, derived from the study in which responses in NHP can specifically be boosted by addition of PAgs to protein subunit vaccines are needed (101).

Therefore, clinical trials targeting γδ T cells may offer improved outcomes that can best be harnessed for immunotherapy approaches, as widely experienced in cancer immunotherapy (102).

### HLA-E–Restricted T Cells

HLA-E has been classically defined by the ability to present signal sequence peptides from HLA class I, which inhibit NK cells cytolytic activity upon interaction with CD94/NKG2A receptors (103). However, it has been shown that HLA-E molecules are able to bind and present other self or pathogen-derived peptides, including *M. tuberculosis*, and can be recognized by adaptive T cells (104–106) (Figure 1).

HLA-E/mycobacterial peptide complexes are recognized differently from HLA-E/self-peptides; in fact, in our previous study, we have demonstrated that the latter are predominantly recognized by NK cells in a CD94 dependent manner; the former are specifically recognized by CD8^+^ T cells in a CD3/TCR αβ-dependent manner (106).

The binding of peptides to HLA-E molecule has been described (107). The peptide-binding motif reveals that most of the peptides that bind to HLA-E are similar to HLA I leader sequence with P2 Met and PΩ Leu. This motif has been identified for 21 peptides, but the discovery of new HLA-E–specific peptides needs to be characterized in sharing anchor residues or motifs. In fact, the crystal structure analysis of HLA-E- bound to a mycobacterial peptide has revealed that the flexibility of the conformation of the bound peptides is also critical in the activation of CD8 T cells despite the preferred anchor residues (108).

Therefore, HLA-E plays a role in both innate and adaptive immune response, thanks to their interaction with both NK cells and antigen-specific CD8^+^ T cells. One important aspect of HLA-E molecule is its low allelic variability, rendering this molecule an interesting candidate antigen-presenting molecule for peptide-based vaccination strategies (103, 109–111). These T cells can inhibit intracellular *M. tuberculosis* growth in human macrophages. Moreover, compared to class Ia molecules, HLA-E molecule is enriched in *M. tuberculosis* phagosomes and accessible for loading with *M. tuberculosis* peptides generated into the phagosome (112, 113).

Studies in mouse and NHP suggest a contribution of HLA-E–restricted T cells to protective immunity against TB.

It was found that the murine homolog of HLA-E, Qa-1 molecule, can bind and present human HLA-E–binding peptides to murine CD8^+^ T cells, which display cytolytic and regulatory activities (114). Moreover, knockout studies confirmed a direct role for Qa-1 in regulating histopathology and bacterial burden and contributing to protection against *M. tuberculosis* (114).

MHC-E–restricted CD8^+^ T cell responses are elicited in rhesus macaques (Rh) by an experimental rhesus cytomegalovirus (CMV) vaccine, which express genes coding for proteins specific for simian immunodeficiency virus (SIV). This attenuated Rh CMV vaccine showed strong protection against a subsequent challenge with SIV infection, and protection was due to activation of CD8^+^ T cells that recognized SIV peptides bound to either MHC class II or MHC-E molecules, but not conventional MHC class Ia molecules, explaining in part the involvement of MHC-E–restricted T cells in protection (115–117). Further investigation revealed that naturally occurring SIV epitopes matched the Rh CMV vector–elicited CD8^+^ T cell–restricted epitopes contributing to protection against a subsequent SIV challenge (112). Similarly to the model described above, Rh CMV-TB antigen vectors induced strong protection against TB following vaccination in 41% of treated NHPs (118). Moreover, Hansen et al. (118) demonstrated that one of three tested Rh CMV strain 68-1 vectored vaccines expressing six or nine protein from *M. tuberculosis* was able to elicit unconventionally restricted MHC class II and MHC-E restricted CD8^+^ T cell responses. Therefore, viral-vectored vaccines can be developed in order to induce immunogenicity of HLA-E–restricted T cells against many pathogens in human patients.

Recently, we have demonstrated that *M. tuberculosis*–specific and HLA-E–restricted CD8^+^ T cells are abundant but exhausted in peripheral blood of TB–HIV-1–coinfected patients, and this dysfunctional phenotype is correlated with high levels of PD-1 molecule expression. The use of anti-PD1 mAb may restore, even partially, the number and the functions of *M. tuberculosis*–specific and HLA-E–restricted CD8^+^ T cells (119). Like NKT and MAIT cells, HLA-E–restricted CD8^+^ T cells are associated with exhaustion. This abnormal phenotype is probably caused by the direct recognition of *M. tuberculosis*–infected cells and the exposure to high levels of inflammatory cytokines. Further research is needed to develop strategies for restoring this subset in patients with *M. tuberculosis* infection/disease, with or without HIV coinfection, in order to better define the therapeutic potential of immune checkpoint blockade.

Taking advantage for the relative monomorphism of HLA-E molecule and for its stable expression in HIV–*M. tuberculosis* infection, which represents an important issue of global health, the use of antigen recognition through HLA-E molecule, should be considered as another valuable approach to promote or boost activation of CD8^+^ T cells in vaccine formulation or immunotherapy.

## Perspective

Translational research can begin to use the knowledge of unconventional T cell biology to develop new immunotherapeutic approaches. Vaccine or immunotherapy development could represent a good strategy in infectious diseases prevention, but obviously, it is very important to demonstrate that identified ligands for unconventional T cells are expressed at the surface of infected cells at densities and durations that are able to engage TCRs and induce T cell activation and immunological memory phenotype. In fact, the efficacy of vaccine or immune protection in case of the re-encounter with the same pathogen could be reached by the induction of memory T cells.

Therefore, unconventional T cells can be used to improve T cell immunotherapy, thanks to several aspects, such as the rapid cytokine release without the need to previous clonal expansion due to their presence at high number of available experienced antigen-specific cells that have developed as memory-like state (77). Another important aspect is represented by the monomorphic model of antigen recognition by unconventional T cells that could be universally effective in human infectious diseases and cancer context. Moreover, their TCRs will not be able to give alloreactive responses and to cause GVHD, making these cells more suitable in cellular therapy such as chimeric antigen receptor (CAR) T cell therapy (120, 121).

MAIT, NKT, and γδ T cells are crucial players in the development and maintenance of immunity. These aspects have been demonstrated by the array of infectious, inflammatory, and malignant diseases in which they play diverse roles (81, 122–127).

Depending on the nature of the infectious or inflammatory setting, these can range from host protective functions, for example, antimicrobial or antitumor responses, to the augmentation of disease (122–126). A number of clinical trials based on γδ T cell therapy have been conducted or are ongoing to evaluate the safety and antitumor efficacy (128). Moreover, several clinical trials have assessed the safety and efficacy of Vγ9Vδ2 T cells for immunotherapy. Because of their high plasticity, studies using CAR- γδT cells could be of great interest also in infectious diseases.

Until now, no clinical trials have investigated on the efficacy of unconventional T cells in inducing protection toward *M. tuberculosis* disease.

Because of the role that these cellular populations play during the early stages of infection, they could be studied as promising tools in immunotherapy against the intracellular pathogen.

Finally, therapy using anti–PD-1 and anti–CTLA-4 monoclonal antibodies, important checkpoints of the immune response, has demonstrated to play an important and valid approach to treat some types of cancer (129), where it is assumed primarily to enhance CD8^+^ T cell–mediated tumor destruction.

Given that unconventional T cells also express the inhibitory receptors such as PD-1 upon activation and that blockade of these receptors can enhance their effector activities and antitumor capacity (74, 130–132), the potential role that unconventional T cells play should be considered in infectious disease and the immunotherapy for infectious diseases, and the incorporation of unconventional T cells into these studies has the potential to provide novel approaches to this important area of medicine.

## Data Availability Statement

The original contributions presented in the study are included in the article/supplementary material, further inquiries can be directed to the corresponding author/s.

## Author Contributions

FD and NC wrote the manuscript. All authors have reviewed the manuscript and have made intellectual contributions to the work.

## Conflict of Interest

The authors declare that the research was conducted in the absence of any commercial or financial relationships that could be construed as a potential conflict of interest.
